# Treatment of Tetanus

**Published:** 1918-01

**Authors:** 


***Treatment of Tetanus.*** (Extract from a pamphlet published by the Under-Secretary of State for the Service de Santé, entitled "Practical Suggestions Concerning Certain Infectious Diseases".) Trans. from the *Archives de Médecine et de Pharmacie Militaires*, Sept., 1916, p. 356.

*Preventive Treatment.* — *Who should receive injections of serum?* Every man with even a small wound which has been contaminated with earth, especially if the wound is irregular, or if it contains projectiles or bits of clothing. Two injections of serum should be administered. Under present conditions wounds which do not require this prophylaxis are exceptional.

*When ought the injection to be made?* As quickly as possible after the injury. The second preventive injection should be made from 5 to 6 days after the first.

*Doses of Serum.* — Injections should be made subcutaneously in the flank, in the neighborhood of the tenth rib. The first should be a dose of 10 cc. and the second from 10 cc. to 20 cc., according to the wound.

*Reinjections.* — *When should serum be reinjected?* Immediately upon the appearance of the first symptom of tetanus.

If an operation is necessary, even several months after the injury, an injection of 10 cc. should be administered both before and after the operation.

A patient whose wound is slow in healing should receive an injection of 10 cc. every 10 days until the healing is completed.

*Treatment of Established Tetanus.* — *The use of serum.* Treatment differs accordingly as tetanus sets in a few days after the injury, or several months later.

If it sets in within a few days of the injury, begin by an injection into the spinal column of 40 cc. of serum, administered very slowly; the following day inject subcutaneoulsy 30 cc. to 50 cc. of serum.

In late tetanus, only subcutaneous injections should be given, in the same doses mentioned above; in such a case, cleansing of the wound and the extraction of foreign bodies may be attempted; such an operation is a prophylactic measure that must not be neglected.

*Sedative Treatment of the Nervous System.* — The patient should be placed in a separate room and shielded from all noise, jarring, bright light, and all sensory excitement.

Chloral hydrate may be administered locally (8 to 10 gr. in 24 hours), or in the form of a subcutaneous injection (6 to 8 gr. in a 2 o/o solution, in 24 hours).

Inject intravenously, if necessary, 1/4 mgr. of adrenalin 4 times every 24 hours.

If violent contractions take place during the day, it is possible to relieve the patient temporarily by injecting subcutaneously 10 cc. of a 40 o/o solution of sulphate of magnesia. In urgent cases the injection may be made in the spinal column.

Bacelli's method of treatment is to inject subcutaneously from 1 to 2 gr. of carbolic acid several times during the 24 hours.

| | |
|---|---|
| Phenic acid. . . . . . . . . . . . . . . . . . . . | 2 gr. |
| Glycerine. . . . . . . . . . . . . . . . . . . . | 5 gr. |
| Water.. . . . . . . . . . . . . . . . . . . . . | 93 gr. |

At first, small injections of 0.15 gr. to 0.20 gr. of carbolic acid are administered (7 cc. to 10 cc. of the solution). The patient's urine must be watched with care. Since these injections produce the sensation of burning, they must be made slowly.

